# Antifungal susceptibility testing of *Candida* species isolated from the immunocompromised patients admitted to ten university hospitals in Iran: comparison of colonizing and infecting isolates

**DOI:** 10.1186/s12879-017-2825-7

**Published:** 2017-11-21

**Authors:** Parisa Badiee, Hamid Badali, Teun Boekhout, Kambiz Diba, Abdolkarim Ghadimi Moghadam, Ali Hossaini Nasab, Hadis Jafarian, Rasoul Mohammadi, Hossein Mirhendi, Mohammad Javad Najafzadeh, Ahmad Shamsizadeh, Jafar Soltani

**Affiliations:** 1 Prof. Alborzi Clinical Microbiology Research Center, Shiraz University of Medical Sciences, Shiraz, Iran; 2 Department of Medical Mycology and Parasitology/Invasive Fungi Research Centre (IFRC), School of Medicine, Mazandaran University of Medical Sciences, Sari, Iran; 3 Westerdijk Fungal Biodiversity Institute, Utrecht, Netherlands; 4 Institute for Biodiversity and Ecosystem Dynamics (IBED), University of Amsterdam, Amsterdam, Netherlands; 5 Urmia University of Medical Sciences, Urmia, Iran; 6 Department of Pediatrics, Yasuj University of Medical Sciences, Yasuj, Iran; 7 Department of Pediatrics, Kerman University of Medical Science, Kerman, Iran; 8 Department of Medical Parasitology and Mycology, School of Medicine, Infectious Diseases and Tropical Medicine Research Center, Isfahan University of Medical Sciences, Isfahan, Iran; 9 Department of Medical Mycology and Parasitology, School of Public Health and Institute of Health Research, Tehran University of Medical Sciences, Tehran, Iran; 10 Department of Parasitology and Mycology, School of Medicine, Mashhad University of Medical Sciences, Mashhad, Iran; 11 Infectious and Tropical Diseases Research Center, Health Research Institute, Ahwaz Jundishapur University of Medical Scienses, Ahvaz, Iran; 12 Department of Pediatrics, Besat Tertiary Hospital, Kurdistan University of Medical Sciences, Sanandaj, Iran

**Keywords:** Colonizing *Candida*, *Candida* infected patients, *Candida* wild-type, *Candida* susceptibility testing, *Candida Albicans*

## Abstract

**Background:**

Antifungal susceptibility testing is a subject of interest in the field of medical mycology. The aim of the present study were the distributions and antifungal susceptibility patterns of various *Candida* species isolated from colonized and infected immunocompromised patients admitted to ten university hospitals in Iran.

**Methods:**

In totally, 846 *Candida* species were isolated from more than 4000 clinical samples and identified by the API 20 C AUX system. Antifungal susceptibility testing was performed by broth microdilution method according to CLSI.

**Results:**

The most frequent *Candida* species isolated from all patients was *Candida albicans* (510/846). The epidemiological cutoff value and percentage of wild-type species for amphotericin B and fluconazole in *Candida albicans*, *Candida tropicalis*, *Candida glabrata* and *Candida krusei* were 0.5 μg/ml (95%) and 4 μg/ml (96%); 1 μg/ml (95%) and 8 μg/ml (95%); 0.5 μg/ml (99%) and 19 μg/ml (98%); and 4 μg/ml (95%) and 64 μg/ml (95%), respectively. The MIC90 and epidemiological cutoff values to posaconazole in *Candida krusei* were 0.5 μg/ml. There were significant differences between infecting and colonizing isolates of *Candida tropicalis* in MIC 90 values of amphotericin B, and isolates of *Candida glabrata* in values of amphotericin B, caspofungin, and voriconazole (*P* < 0.05).

**Conclusions:**

Our findings suggest that the susceptibility patterns of *Candida* species (colonizing and infecting isolates) in immunocompromised patients are not the same and acquired resistance was seen in some species.

## Background

Antifungal susceptibility patterns of infectious fungi are a crucial determinant that contributes to the outcome of patients. While the incidence of *Candida* infections is increasing, the choice of suitable antifungal agents is limited due to the resistance of some species to several antifungals. *Candida* species can cause superficial to life-threatening candidemia and hospital-acquired infections in humans [1, 2]. *Candida albicans* remains the leading *Candida* species that causes infection, but the epidemiology of non-*albicans Candida* species has been on the rise [3, 4]. These species cause infections in patients, especially those with underlying diseases. The activities of antifungal agents are important therapeutic options to control infections caused by these yeasts. The appropriate treatments are dependent on the immune status and underlying diseases of patients, the specific *Candida* species involved and its susceptibility pattern to antifungal agents.

The Clinical and Laboratory Standards Institute (CLSI) developed new *Candida* species-specific clinical breakpoints for some antifungal agents, like fluconazole, voriconazole, and echinocandins [5, 6]. Use of such breakpoints can change the previously known *Candida* species sensitivity impact patterns and consequently the management of the patients.

A few multicenter surveillance studies have been conducted comparing antifungal susceptibility patterns of isolates obtains from the infected (INFECT) and colonized (COL) hospitalized patients. Therefore, in the present study, the distributions and antifungal susceptibility patterns of various *Candida* species isolated from infected and colonized immunocompromised patients admitted to 10 university hospitals in Iran were reported using CLSI species-specific clinical breakpoints and epidemiological cutoff values (ECV).

## Methods

### Study design and patients

The present study is a cross-sectional study carried out during 2014-2015 in patients admitted to 10 university hospitals in Iran. The participant university hospitals were as follows: Ahvaz, Isfahan, Kerman, Mashhad, Sanandaj, Sari, Shiraz, Tehran, Urmia, and Yasuj. *Candida* species isolated were divided into infecting and colonizing isolates. Infecting *Candida* species were isolated from various clinical samples, like blood, cerebrospinal fluid, bronchoalveolar lavage, and sputum of the infected patients according to European Organization for Research and Treatment of Cancer/Invasive Fungal Infections Cooperative Group and the National Institute of Allergy and Infectious Diseases Mycoses Study Group criteria [7]. Colonizing species were isolated from the oral cavity, urine, nose and swab rectum of immunocompromised hospitalized patients without any clinical signs and symptoms of *Candida* infections. The underlying diseases in patients were a solid organ and bone marrow transplantation, hematologic disorders including acute lymphoblastic leukemia, chronic lymphocytic leukemia, acute and chronic myeloid leukemia, aplastic anemia, pancytopenia, Burkitt lymphoma; Rhabdomyosarcoma and histiocytosis.

Species identification and antifungal susceptibility testing of the isolates were performed at Professor Alborzi Clinical Microbiology Research Center, Shiraz University of Medical Sciences, Shiraz, Iran. All samples were cultured on sabouraud dextrose agar (Merck, Germany) at room temperature and all isolates were subcultured on potato dextrose agar (OXOID LTD, Basingstoke, Hampshire, England) twice for 48 h at 35 °C to check the purity of the colonies. Species identification was confirmed by germ tube and chlamydospore production tests, and API 20 C AUX system (bioMerieux, Swiss), according to the manufacturer’s instructions.

### Antifungal susceptibility studies

Susceptibility values to amphotericin B (AMB), fluconazole (FLU), voriconazole (VOR), itraconazole (ITR), and posaconazole (POS) were assessed by the CLSI broth microdilution methods M27-A3 and M27-S4 [5, 8]. Two reference strains, *C. parapsilopsis* ATCC 22019 and *C. krusei* ATCC 6258, were included in each test as quality control isolates.

Powders of AMB and POS (Sigma, Germany), FLU, ITR, VOR, and CAS (Sigma, USA) were obtained from the respective manufacturers. RPMI 1640 (Sigma, St. Louis, Missouri) was made according to the manufacturer’s protocol and buffered to pH 7.0 with 0.165 N-morpholino propanesulfonic acid (MOPS) buffer (Sigma, USA). Stock solutions with 10-fold concentration for each antifungal were prepared in dimethyl sulfoxide (DMSO). The final concentrations of the working solutions were obtained by using RPMI medium. The final concentrations of the antifungal agents were 0.032 to 16 μg/ml for AMB, ITR, POS and VOR 0.125 to 64 μg/ml for FLU, and 0.016-8 μg/ml for CAS. The inoculum suspensions (0.5 McFarland) were prepared by the spectrophotometric method (at 530 nm) (Pharmacia biotech Cambridge, England ultrospec 3000 UV/visible spectrophotometer), and diluted to 0.5 × 10^3^ or 2.5 × 10^3^ cells/ml using RPMI 1640 medium. A 100-μl volume of yeast inoculum and an equal volume of antifungal agents were added to each well. Drug-free and yeast-free wells were included as positive and negative controls. The MIC of AMB was reported as the lowest drug concentration that complete inhibition of any discernible growth (100%) and for FLU, ITR, VOR, POS, and CAS the lowest concentration that inhibits 50% of the growth, compared to positive controls was taken.

### Data collection and statistical analysis

Data were collected in WHONET version 5.6 database and SPSS version 16 (SAS Institute, Cary, NC, USA). The comparison of antifungal susceptibility rates between INFECT and COL species was made using student T-test and Mann-Whitney U tests. *P* < 0.05 was considered significant.

## Results

More than 4000 samples from different sites of patients were examined and 846 *Candida* species were isolated. Sample site and distribution of cultured *Candida* species isolated from the immunocompromised patients were presented in Table 1. The INFEC species were isolated from bronchoalveolar lavage, blood, fluid (joint, abdominal fluid, peritoneal fluid), abscess, sputum, and COL species from mouth, nose, rectum, urine, and vagina. The most frequent *Candida* species isolated from the patients was *C*. *albicans* (Table 2)*.* The rates of the other *Candida* species were: *C. tropicalis* 74 (8.8)*, C*. *glabrata* 71 (8.3%), *Candida famata* 48 (5.7%), *C*. *parapsilopsis* 47 (5.6%), *Candida kefyr* 38 (4.5%), *Candida krusei* 23 (2.7%), *Candida dubliniensis* 13 (1.5%) and *C. intermedia, Candida lusitaniae* and *Candida guilliermondii* 22 (2.6%).

**Table 1:** Sample site and distribution of cultured *Candida* species isolated from the immunocompromised patients

Sample Site	Distribution of *Candida* species
Bronchoalveolar lavage	*Candida albicans, Candida tropicalis, Candida famata, Candida parapsilopsis, Candida kefyr*
Blood	*Candida albicans, Candida tropicalis, Candida glabrata, Candida famata, Candida parapsilopsis, Candida kefyr*
Fluid^a^	*Candida albicans, Candida parapsilopsis*
Abscess	*Candida albicans*
Sputum	*Candida albicans, Candida glabrata, Candida famata, Candida kefyr, Candida dubliensis, other*
Mouth	*Candida albicans, Candida tropicalis, Candida glabrata, Candida famata, Candida parapsilopsis, Candida kefyr, Candida dubliensis, other*
Nose	*Candida albicans, Candida tropicalis, Candida famata, Candida parapsilopsis*
Rectum	*Candida albicans, Candida tropicalis, Candida glabrata, Candida famata, Candida kefyr, Candida dubliensis, others*
Urine	*Candida albicans, Candida tropicalis, Candida glabrata, Candida famata, Candida parapsilopsis, Candida kefyr*
Vagina	*Candida albicans, Candida tropicalis, Candida glabrata, Candida famata, Candida parapsilopsis, Candida kefyr, Candida dubliensis, other*

^a^Fluid include: Joint, abdominal fluid, peritoneal fluid

**Table 2:** Distribution of *Candida* species isolated from the colonized and infected patients

*Candida* spp.	Colonized isolates Number/%	Invasive isolates Number/%	Total
*Candida albicans*	237 (56.3%)	273(64.2%)	510 (60.3%)
*Candida tropicalis*	36 (8.6%)	38 (8.9%)	74 (8.8%)
*Candida glabrata*	53(12.6%)	18 (4.7%)	71(8.3%)
*Candida famata*	28 (6.7%)	20 (4.6%)	48 (5.7%)
*Candida parapsilosis*	12 (2.9%)	35 (8.2%)	47 (5.6%)
*Candida kefyr*	18 (4.3%)	20 (4.7%)	38(4.5%)
*Candida krusei*	13 (3%)	10 (2.4%)	23(2.7%)
*Candida dubliniensis*	10 (2.3%)	3 (0.7%)	13(1.5%)
Others^a^	14 (3.3%)	8 (1.9%)	22 (2.6%)
Total	421	425	846

^a^Others included: *Candida intermedia, Candida lusitaniae* and *Candida guilliermondii*

The susceptibility patterns of COL and INFEC isolates to six antifungal agents are shown in Table 3. In INFEC and COL isolates, the MIC90 values for AMB in *C. albicans* (0.25 μg/ml and 0.25 μg/ml), *C. parapsilosis* (0.032 μg/ml, and 0.25 μg/ml), and *C. famata* (0. 25 μg/ml and 0.25 μg/ml) did not differ significantly (*P* > 0.05). The MIC90 values of AMB in *C. tropicalis* and *C. glabrata* in INFEC and COL isolates were 4 μg/ml and 0.125 μg/ml; and 8 μg/ml and 0.064 μg/ml, respectively (*P* < 0.05). The MIC90 values of *C. krusei* in INFECT and COL isolates to AMB were 8 μg/ml and 4 μg/ml, respectively, with an ECV of 4 μg/ml. The resistance rates for FLU in INFEC isolates of *C. albicans*, *C. tropicalis*, *C. glabrata*, and *C. parapsilosis* were 4.9% (12/273), 10.5% (4/38), 11.1% (2/18) and 2.9% (1/35), respectively. The resistance rates for INFECT and COL isolates in *C. albicans* and *C. krusei* to ITR were 12.7% (35/273) and 2.6% (7/273); and 33.3% (3/10) and 20% (2/10), respectively.

**Table 3:** In vitro suseptibility patterns of *Candida* species isolates from Colonized (C) and Infected (I) patients

Species Antifungal	I Ra	I R%	I GM	I MIC_90_ (μg/ml)	C Ra	C R%	C GM	C MIC_90_ (μg/ml)	Total ECV (μg/ml)	Total Wild type (%)	Total Non-wild type (%)
*Candida albicans*											
AMB	0.032-16	3.3	0.052	0.25	0.032-32	0.9	0.039	0.25	0.5	≤ 0.5(95%)	> 0.5(5%)
CAS	0.032-1	0.5	0.03	0.25	0.032-1	0.4	0.041	0.125	0.25	≤ 0.25(98%)	> 0.25(2%)
VOR	0.032-2	6.9	0.032	0.125	0.032-16	5.4	0.035	0.064	1	≤ 1(97%)	> 1(3%)
FLU	0.032-64	4.9	0.254	2	0.032-64	0.5	0.254	2	4	≤ 4(96%)	> 4(4%)
POS^a^	0.032-8	–	0.044	0.25	0.032-4	–	0.031	0.032	0.25	≤ 0.25(96%)	> 0.25(4%)
ITR	0.032-16	12.7	0.104	1	0.032-16	2.6	0.049	0.125	1	≤ 1(98%)	> 1(2%)
*Candida tropicalis*											
AMB	0.032-8	18.4	0.115	4	0.032-0.5	0	0.033	0.125	1	≤ 1(95%)	> 1(5%)
CAS	0.032-0.5	0	0.041	0.25	0.032-4	2.9	0.046	0.125	0.5	≤ 0.5(99%)	> 0.5(1%)
VOR	0.032-16	14.3	0.056	1	0.032-16	8.3	0.033	0.125	1	≤ 1(96%)	> 1(4%)
FLU	0.032-64	10.5	0.361	4	0.064-64	8.8	0.302	2	4	≤ 4(95%)	> 4(5%)
POS	0.032-0.25	–	0.029	0.25	0.032-16	–	0.035	0. 125	0.25	≤ 0.25(95%)	> 0.25(5%)
ITR	0.032-2	13.2	0.1	1	0.032-16	0	0.078	0.5	1	≤ 1(96%)	> 1(4%)
*Candida glabrata*											
AMB	0.032-16	11.1	0.101	8	0.032-0.5	0	0.031	0.064	0.5	≤ 0.5(99%)	> 0.5(1%)
CAS	0.032-8	22.2	0.086	4	0.032-0.5	9.8	0.113	0.5	0.5	≤ 2 (96%)	> 2 (4%)
VOR	0.16-4	0	0.088	2	0.032-0.5	0	0.05	0. 25	0.5	≤ 0.5 (96%)	> 0.5(4%)
FLU	0.25	11.1	1.17	16	0.064-16	0	0.842	4	16	≤ 16 (98%)	> 16(2%)
POS	0.032-16	–	0.179	8	0.032-16	–	0.082	0.5	4	≤ 4 (95%)	> 4(5%)
ITR	0.032-16	77.8	0.739	4	0.032-16	15	0.233	1	2	≤ 2 (96%)	> 2(4%)
*Candida famata*											
AMB	0.032-2	5	0.038	0.25	0.032-1	0	0.037	0.25	0.25	≤ 0.25(95%)	> 0.25(5%)
CAS	0.032-16	0	0.043	0.5	0.032-0.25	0	0.035	0.25	0.25	≤ 0.25(95%)	> 0.25(5%)
VOR	0.032-0.5	0	0.024	0.5	0.032-1	0	0.034	0.125	0.25	≤ 0.5(98%)	> 0.5(2%)
FLU	0.064-8	0	0.278	0.5	0.032-8	0	0.268	0.5	1	≤ 1(95%)	> 1(5%)
POS	0.032-1	–	0.026	0.5	0.032-0.5	0	0.031	0.064	0.5	≤ 0.5(98%)	> 0.5(2%)
ITR	0.032-1	10	0.081	0.5	0.032-1	4.3	0.062	0.5	0.5	≤ 0.5(98%)	> 0.5(2%)
*Candida parapsilopsis*											
AMB	0.032-0.5	0	0.023	0.032	0.032-0.25	0	0.027	0.032	0.25	≤ 0.25(95%)	> 0.25(5%)
CAS	0.032-0.25	0	0.037	4	0.032-4	0	0.283	0.125	0.125	≤ 4(98%)	> 4(2%)
VOR	0.032-0.032	0	0.017	0.032	0.032-0.25	0	0.025	0.032	0.032	≤ 0.032(96%)	> 0.032(4%)
FLU	0.064-8	2.9	0.402	4	0.064-2	0	0.298	2	2	≤ 2(100%)	> 2(00%)
POS	0.032-0.032	–	0.017	0.032	0.032-0.5	–	0.03	0.032	0.032	≤ 0.032(96%)	> 0.032(4%)
ITR	0.032-0.5	0	0.0102	0.5	0.032-0.032	0	0.02	0.125	0.5	≤ 0.5(100%)	> 0.5(00%)
*Candida kefyr*											
AMB	0.032-1	0	0.045	1	0.032-1	0	0.03	0.064	1	≤ 1(99%)	> 1(1%)
CAS	0.032-0.125	0	0.028	0.25	0.032-2	0	0.031	0.25	0.125	≤ 0.25(95%)	> 0.25(5%)
VOR	0.032-0.032	0	0.018	0.032	0.032-0.125	0	0.021	0.032	0.032	≤ 0.032(96%)	> 0.032(4%)
FLU	0.25-0.5	0	0.397	1	0.064-2	0	0.185	0.5	1	≤ 1(96%)	> 1(4%)
POS	0.032-0.032	–	0.018	0.032	0.032-0.125	–	0.021	0.032	0.032	≤ 0.032(96%)	> 0.032(4%)
ITR	0.032-0.125	0	0.032	0.125	0.032-0.25	0	0.037	0.125	0.125	≤ 0.125(96%)	> 0.125(96%)
*Candida krusei* ^*b*^											
AMB	0.032-8	40	1.004	8	0.032-4	40	0.386	4	4	≤ 4(95%)	> 4(5%)
CAS	0.032-2	30	0.2	2	0.032-0.5	0	0.092	0.25	2	≤ 2(99%)	> 2(1%)
VOR	0.032-16	20	0.284	2	0.032-16	7.7	0.235	0.5	2	≤ 2(95%)	> 2(5%)
FLU	2-64	–	17.9	64	0.25-64	–	6.817	64	64	≤ 64(95%)	> 64(5%)
POS	0.032-0.5	–	0.126	0.5	0.032-16	–	0.214	0.5	0.5	≤ 0.5(95%)	> 0.5(5%)
ITR	0.064-1	33.3	0.2	2	0.064-16	15.4	0.346	1	2	≤ 2(95%)	> 2(5%)
Other *Candida* spp.^c^											
AMB	0.032-0.032	0	0.032	0.032	0.032-1	0	0.032	0.064	0.064	≤ 0.064(95%)	> 0.064(95%)
CAS	0.032-0.064	0	0.021	0.064	0.032-1	0	0.064	0.064	0.064	≤ 0.064(95%)	> 0.064(95%)
VOR	0.032-0.032	0	0.032	0.032	0.032-0.125	0	0.026	0.032	0.125	≤ 0.125(100%)	> 0.125(100%)
FLU	0.0125-4	0	0.33	4	0.064-8	0	0.227	0.5	4	≤ 4(95%)	> 4(95%)
POS	0.032-0.032	0	0.032	0.032	0.032-0.064	0	0.023	0.032	0.032	≤ 0.032(95%)	> 0.032(95%)
ITR	0.032-0.0125	0	0.024	0.125	0.032-0. 25	0	0.042	0.25	0.25	≤ 0.25(100%)	> 0.25(100%)

*Ra* Range, *MIC* minimum inhibitory concentration, *R* resistant, *MIC*_90_ Lowest concentration at which 90% of the isolates are inhibited

^a^Posaconazole has no breakpoint in new CLSI

^b^Isolates of *C. krusei* are considered resistant to fluconazole, irrespective of the MIC

^c^Others: include; *C. intermedia, C. dubliniensis, C. lusitaniae* and *M. guilliermondii* (*C. guilliermondii*)

Resistance rates to VOR in the INFEC isolates of *C*. *albicans*, *C*. *tropicalis*, and *C. krusei* were 6.9% (19/273), 14.3% (5/38) and 20% (2/10), and in COL isolates 5.4% (13/237), 8.3% (3/36) and 7.7% (1/13), respectively, without significant differences in MIC values (*P* > 0.05). The MIC90 values of POS for all species were < 0.5 μg/ml, except in INFECT *C*. *glabrata* isolates that showed a MIC90 value of 8 μg/ml. The ECV and MIC 90 values for FLU in *C. krusei* were both 64 μg/ml in groups with GM 17.9 and 6.817 in INFEC and COL isolates, respectively. Susceptible dose dependence for ITR in *C. albicans*, *C. krusei*, and *C. kefyr* in INFECT and COL isolates were 35.3% and 24.2%; 33.3% and 69.2%; and 16.7% and 22.2%, respectively. Also, 72.9% of COL *C*. *glabrata* were susceptible dose dependent to ITR. The ECV for this antifungal agent for all *Candida* species was ≤ 1 μg/ml, except *C*. *glabrata* which were 2 μg/ml. The MIC90 values for CAS in all *Candida* isolates ranged between 0.25 μg/ml and 0.5 μg/ml, except in INFECT isolates of *C*. *glabrata* and *C. parapsilosis* (4 μg/ml), and for both group isolates of *C. krusei* a MIC90 of 2 μg/ml was observed.

**Fig. 1:**
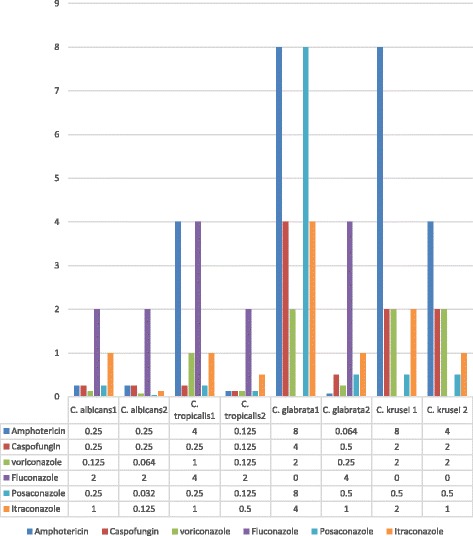
Comparison of MIC90 values in some *Candida* species in Infecting (1) and Colonizing (2) isolates

The comparison of MIC90 values for antifungal agents in INFECT and COL isolates are shown in Fig. 1. There was no significant difference between COL and INFECT *C. albicans*, *C. famata, C. kefyr*, *C. krusei*, *C. intermedia, C. dubliniensis, C. lusitaniae* and *C. guilliermondii* isolates in all antifungal agents in this study (*P* > 0.05). However, a significant difference between INFECT and COL isolates of *C. tropicalis* in MIC90 values of AMB (*P* < 0.05) was observed. *Candida krusei* INFECT and COL isolates presented high ECV and MIC90 values for all antifungal agents, except POS. As for *C. glabrata*, there were significant differences between INFECT and COL isolates in AMB, CAS, and VOR (*P* < 0.05), and MIC90 values in isolates from both groups for FLU, POS, and ITR that were higher than those for other *Candida* species.

## Discussion

The most frequent *Candida* species isolated from the patients was *C*. *albicans* which occurred more in INFEC isolates due to its pathogenic mechanism factors [9]. The rates of *Candida* species involved in infections differ in the literature, but in many studies, *C. albicans* is the most prevalent species [10–12]. The second most frequent *Candida* species in this study was *C. tropicalis.* In a study from one center in Iran, the second isolated species from immunocompromised patients was *C. krusei*, while in Indian and Korean populations was *C. parapsilopsis* [10–12]. Distribution of species was found to differ in each region and study population.

In this region, polyenes and azoles are the most common antifungal agents used in the treatment of patients with fungal infections. In this study, the MIC90 values of AMB were significantly different (*P* < 0.05) in *C. tropicalis* and *C. glabrata* INFEC and COL isolates. The highest MIC90 values to AMB belonged to *C. krusei* INFECT and COL isolates. In Castanheira et al. the MIC90 value for AMB was reported to be 1 μg/ml in all *Candida* isolates, except *C. krusei* which had a MIC of 2 μg/ml [13]. In the present study, resistance rates to AMB in INFECT isolates in *C. albicans* were (3.3%, 9/273), *C. tropicalis* (18.4%, 7/38), *C. glabrata* (11.1%, 2/18), and *C. krusei* (40%, 4/10), whereas in COL isolates resistant rates were only seen in *C. albicans* (0.9%, 3/273) and *C. krusei* (40%, 4/10) (Table 3). These rates were reported in INFECT *C. albicans*, *C. tropicalis, C. glabrata* and *C. krusei* isolate as 7% (12/172), 33.3% (2/6), 15% (6/40) and 10% (6/60), respectively [11]. There have been few studies about susceptibility patterns of *Candida* species in colonized patients. In Haddadi et al. the resistance rates of COL isolates to AMB in *C. albicans*, *C. glabrata* and *C. krusei* were reported to be 3% (4/117), 7.5% (1/14), and 27.7% (5/18), respectively [14]. The MIC 90 value for AMB in 1310 isolated *C. albicans* in Castanheira et al. was reported 1 μg/ml which is higher than of our study (0.25 μg/ml) [15].The results of the resistance rates and MIC90 value in the present study for INFECT and COL isolates were lower than that in other studies, due to their study population, and limited use of antifungal agents in some cities. Unfortunately, in our region, fungal infections are not clear for some clinicians and treatment and prophylaxis for it is not routine in some health care systems.

During the past few years, the CLSI have adjusted the breakpoints for FLU, VOR, and ITR and new breakpoints were reported [5]. In the present study, the most resistant species to FLU in INFEC isolates of the *Candida* species were *C. glabrata* and *C. tropicalis*. Compared with other *Candida* species, *C. krusei* and *C*. *glabrata* are generally documented as the causes of invasive candidiasis with reduced susceptibility to FLU. The MIC 90 values for FLU in *C. albicans* and *C. glabrata* in our study were 2 μg/ml and 16 μg/ml in the two groups, respectively. The MIC90 values for FLU in Castanheira et al. in clinical *C. albicans* and *C. glabrata* isolates were reported as 0.25 μg/ml and 32 μg/ml with resistance rates of 0.3 and 7.9%, respectively [13]. The resistance rate to FLU was reported 2.6% in infected patients in Korea [10]. In Pfaller et al. “resistance to fluconazole was seen in 0.5% of *C. albicans* isolates, 11.1% of *C. glabrata* isolates, 2.5% of *C. parapsilosis* isolates, 4.5% of *C. tropicalis* isolates, and 20.0% of *C. guilliermondii* isolates” [16]. In the present study, the resistance rates to ITR in *C. glabrata* INFEC and COL isolates were 77.8% (14/18) and 15% (8/53), respectively (*P* < 0.05). This resistance rate in INFECT was similar to the results obtained in immunocompromised patients in our previous study (i.e. 85.5%) [11]. Resistance to ITR was reported in many studies [11, 14, 17]. In Haddadi et al., this rate was 22.5% (49/217) in all COL isolates [14]. In Cuenca-Estrella et al. resistance of all *Candida* species to ITR was seen, especially in *C. glabrata* [18]. Strains susceptible dose dependent to ITR were reported in many studies [11, 14, 17]. Susceptible dose dependence in the present study for ITR was seen in many *Candida* species, especially COL *C*. *glabrata* isolates. Itraconazole and FLU are the most commonly prescribed azole agents in our region, which may explain the increased resistance rates and susceptible dose dependence observed for these antifungal agents in our study. The presence of many susceptible dose dependent isolates may suggest the future emergence of these species with resistant isolates.

Cross-resistance of VOR and other azoles, such as FLU and ITR, can occur due to previous exposure. Resistance rates to VOR in the INFEC and COL isolates of *Candida* species were observed without significant differences in MIC values. The ECV of VOR for *C. glabrata* according to the new CLSI breakpoint is 0.5 μg/ml. The MIC90 for *C. glabrata* INFECT isolates in the present study was 2 μg/ml, indicating that most of the isolates were non-WT. Voriconazole MICs of > 0.12 μg/ml were reported among *C. glabrata* and *C. krusei* isolates in Cuenca-Estrella M et al. [18].

According to the newly defined CLSI breakpoints, there is no breakpoint defined for POS. In the present study, the MIC90 of POS for INFECT *C*. *glabrata* was 8 μg/ml and other *Candida* species had < 0.5 μg/ml MIC90 value. A MIC value above 8 μg/ml for POS was reported in Soczo et al. for *C. albicans* and *C. glabrata* isolates that were resistant to FLU [19]. Also, a POS MIC90 value of 0.06 μg/ml was reported in *C. albicans* and 2 μg/ml for *C. glabrata* isolates in Castanheira et al. [13]. In Mahmoudabadi et al. 94% of the isolates were inhibited by POS at < 2 μg/mL after 24 h, whereas 6% of isolates had MICs of > 4 μg/mL [20]. According to the present study, the ECV and MIC90 values of POS in *C. krusei* were 0.5 μg/ml, and POS is the best antifungal agent for the treatment of infections due to this species. POS is an expensive antifungal in Iran and has a restricted use, which may contribute to the observed low MIC90 value.

In Iran, caspofungin has more usage than other echinocandin antifungal agents. In the current study, MIC90 values for CAS in INFECT *C*. *glabrata* and *C. parapsilosis* and for both groups of isolates of *C. krusei* were 4 μg/ml and 2 μg/ml, respectively. The MICs 90 of CAS for *C. krusei* were reported as 4 μg/mL [21, 22]. In the present study, significant differences occurred between the MIC 90 values of CAS in *C. glabrata* COL (0.5 μg/ml) and INFECT (4 μg/ml) isolates and *C. parapsilosis* COL (0.125 μg/ml) and INFECT (4 μg/ml) isolates (*P* < 0.05). Espinel-Ingrof et al. reported that CAS MICs value evaluation for some *Candida* species (such as *C. glabrata* and *C. krusei* according to CLSI breakpoints is not suitable and could lead to reporting an excessive number of wild-type or non-wild type isolates [23]. In Mahmoudabadi et al. the MIC of CAS in 90% of the total INFECT *Candida* isolates was lower than 2 μg/mL [20]. In Abad et al. MIC values for CAS in *C. albicans*, *C. parapsilosis* complex, *C. tropicalis*, *C. glabrata* complex, *C. guilliermondii* and *C. krusei* were reported as 0.008 μg/ml, 2 μg/ml, 0.12 μg/ml, 0.12 μg/ml, 2 μg/ml, and 0.5 μg/ml, respectively [24].

The infections by the other non-*albicans Candida* species (*C. famata*, *C*. *parapsilosis*, *C. kefyr*, *C. dubliniensis*, *C. lusitaniae, C. guilliermondii*, and *C. intermedia*) have been increasing during the past decades. Their susceptibility patterns to antifungal drugs and knowledge of their resistance rates are helpful to the patient management in each region. These non-*albicans Candida* isolated species in this study were susceptible to most antifungal agents. In INFECT isolates, 5 and 10% resistance rates to AMB and ITR were seen in *C. famata*, respectively. *Candida parapsilosis* INFECT isolates were found to have 2.9% resistance to FLU.

According to the ECVs observed in this study, many isolated species in both groups were WT and present an ECV lower than the CLSI breakpoint. Non-WT species were seen most in INFECT isolates. As for *C. glabrata,* there were significant differences between the MIC90 values of INFECT and COL isolates in AMB, CAS, and VOR. Jensen et al. reported treatment ≥ 7 days with azoles following fungal infection can produce resistant species, especially *C. glabrata* that colonizes mucosa [25]. In this study, the population study comprised of immunocompromised humans and all had a history of use of antifungal agents as prophylaxis or treatment while COL isolates were WT without increased MIC values.

## Conclusion

Our work represents the first Iranian multicenter study demonstrating antifungal susceptibility patterns and ECV among INFECT and COL *Candida* species isolates among immunocompromised patients. Our findings suggest that the susceptibility patterns of *Candida* species (COL and INFECT isolates) in patients are not the same. The COL species may be recognized as a reservoir and are important for the management of the patients. Increasing use of antifungal agents needs to be monitored by ongoing national surveillance program.

## Abbreviations

AMB: Amphotericin B
CLSI: The Clinical and laboratory standards institute
COL: Colonized
DMSO: Dimethyl sulfoxide
ECV: Epidemiological cutoff values
FLU: Fluconazole
GM: Geometric mean
INFECT: Infected
ITR: Itraconazole
MOPS: N-morpholino propanesulfonic acid
POS: Posaconazole
Ra: Range
VOR: Voriconazole
WT: Wild-type
